# Validation of the Polish Version of the Keratoconus Outcomes Research Questionnaire: Tool for Vision-Related Quality of Life in Patients with Keratoconus

**DOI:** 10.3390/jcm14092959

**Published:** 2025-04-24

**Authors:** Magdalena Nandzik, Adam Wylęgała, Magdalena Kijonka, Dominika Szkodny, Bartłomiej Markuszewski, Edward Wylęgała

**Affiliations:** 1 Department of Ophthalmology, Faculty of Medical Sciences, Zabrze Medical University of Silesia, 40-760 Katowice, Poland; 2 Department of Ophthalmology, District Railway Hospital in Katowice, Medical University of Silesia, Panewnicka 65, 40-760 Katowice, Poland

**Keywords:** keratoconus, quality of vision, Keratoconus Outcomes Research Questionnaire, psychometric validation, vision-related quality of life

## Abstract

**Background/Objectives:** Keratoconus is a progressive corneal disorder that significantly impairs visual quality and daily functioning. This study aimed to translate, culturally adapt, and validate the Polish version of the Keratoconus Outcomes Research Questionnaire (KORQ), a tool designed to assess keratoconus-related vision problems and their impact on quality of life. **Methods:** This study involved three phases: translation, validation, and optimization. The translation followed cross-cultural adaptation guidelines, including forward translation, back translation, and pretesting. In the validation phase, 100 patients with keratoconus completed the Polish KORQ. Psychometric properties were assessed using Rasch analysis to evaluate item fit, reliability, unidimensionality, and targeting. Exploratory and confirmatory factor analyses (EFA and CFA) were conducted to examine the latent structure of the questionnaire. Regression analysis examined the demographic and clinical factors influencing keratoconus severity and vision-related quality of life, with the total KORQ score as the dependent variable. **Results:** The Polish KORQ demonstrated excellent psychometric properties. Internal consistency was high (α = 0.95 for activity limitation, α = 0.87 for symptoms). Rasch analysis confirmed good item fit and measurement reliability. EFA identified a two-factor structure consistent with the original questionnaire, explaining 53% of the total variance. CFA supported this model, with good fit indices (CFI = 0.981, TLI = 0.980), despite slightly elevated RMSEA (0.104) and SRMR (0.109). The two latent factors were moderately correlated (r = 0.729). Regression analysis showed that uncorrected visual acuity and disease severity significantly predicted lower quality of life. **Conclusions:** The Polish KORQ is a reliable and valid tool for assessing the impact of keratoconus in clinical and research settings.

## 1. Introduction

Keratoconus is a progressive degenerative disorder of the cornea that substantially impairs visual function and diminishes patients’ quality of life [1,2]. The characteristic corneal irregularities associated with keratoconus result in refractive errors, reduced visual acuity, increased light sensitivity, and other visual disturbances [3]. These deficits significantly affect daily activities and psychological well-being, highlighting the necessity for reliable and validated instruments to comprehensively assess the disease’s impact on patients’ lives [4].

Numerous validated instruments for assessing vision-related quality of life are available in Poland, each adapted and validated to ensure cultural and linguistic relevance in measuring the impact of visual impairments on daily functioning [5]. However, these tools do not specifically address the distinct challenges posed by keratoconus. This gap underscores the need for a specialized instrument, such as the Keratoconus Outcomes Research Questionnaire (KORQ), to provide a more accurate evaluation of the unique functional and quality-of-life impairments experienced by patients with keratoconus.

The Keratoconus Outcomes Research Questionnaire (KORQ), developed by Khadka et al. [6] for assessing vision-related quality of life in patients with keratoconus, focusing on the specific visual impairments and symptoms caused by the disease. It comprises 29 items divided into two subscales: activity limitation (18 items), which evaluates the impact of keratoconus on daily activities, such as reading and driving, and symptoms (11 items), which assesses the frequency and severity of visual discomfort. Responses were rated on a four-point scale, with an additional “not applicable” option for items deemed irrelevant by the respondent. The questionnaire is being validated in an increasing number of countries worldwide [7,8,9,10]. It is utilized as a tool for assessing vision quality and monitoring changes, for example, following procedures such as corneal crosslinking [11,12].

This study aims to translate, culturally adapt, and validate the Polish version of the Keratoconus Outcomes Research Questionnaire (KORQ) to ensure its psychometric reliability and validity for assessing vision-related quality of life in Polish patients with keratoconus. It seeks to establish the KORQ as a standardized tool for identifying keratoconus-specific visual and functional impairments while evaluating its performance through regression analysis to determine factors influencing disease severity and visual outcomes in the Polish population.

## 2. Materials and Methods

### 2.1. Study Design

This prospective study was conducted between July 2023 and November 2024 in the Department of Ophthalmology of the District Railway Hospital in Katowice, Poland. This study consisted of three distinct phases: translation, validation, and optimization. During the translation phase, the KORQ questionnaire was translated into Polish, following established guidelines for cross-cultural adaptation. The process began with forward translation of the English version into Polish by two independent native Polish-speaking translators. An interdisciplinary panel reviewed and evaluated the initial translation to ensure linguistic and conceptual equivalence. Subsequently, the Polish version was back-translated into English by another independent native English-speaking translator. The back translation was compared to the original English version by an interdisciplinary panel to resolve discrepancies and confirm the accuracy of the translation. The translated questionnaire was then pretested on 15 patients with keratoconus. During the structured interviews, participants were asked to explain their interpretation of each question, share their initial associations, and paraphrase each question in their own words to verify clarity and comprehensibility. Additionally, they were invited to identify any words or phrases that were confusing, offensive, or otherwise problematic. Based on their feedback, the interdisciplinary team revised the questionnaire to establish its final Polish version.

In the validation phase, a translated questionnaire was administered to 100 patients. The inclusion criteria for participation were as follows: adult men and women with a confirmed diagnosis of keratoconus at any stage and no other significant ophthalmic diseases. Patients using medications that could affect vision were excluded. Patients who had undergone corneal transplantation in one eye were also included, provided that keratoconus was diagnosed in the other eye as well. This approach ensured that the validation process was conducted on a representative group of patients, allowing for a robust assessment of the questionnaire’s psychometric properties and applicability in the Polish clinical context. The psychometric properties of the Polish KORQ were assessed using Rasch analysis. This included evaluating item fit to determine alignment with the Rasch model, assessing reliability to confirm internal consistency, verifying unidimensionality to ensure that each scale measured a single underlying construct, and examining targeting to confirm the items were appropriately matched to the population. Patterns of missing data and response inconsistencies were also analyzed to identify problematic items and evaluate the overall functionality of the questionnaire.

Finally, in the optimization phase, the Polish KORQ was refined based on the results of the validation phase. This re-engineering process involved removing or revising problematic items to enhance the tool’s psychometric properties. Additional testing was conducted to confirm improvements in reliability, validity, and usability, resulting in the final optimized version of the Polish KORQ. This structured approach ensures that the questionnaire is linguistically accurate, culturally relevant, and psychometrically robust.

In addition, each participant underwent a comprehensive ophthalmologic evaluation accompanied by a detailed medical history interview. The assessment included measurements of visual acuity, both corrected and uncorrected, using a Snellen chart. Corneal imaging and analysis were conducted using an Oculus Pentacam HR device (Oculus GmbH, Wetzlar, Germany), enabling the detailed evaluation of corneal parameters.

### 2.2. Statistical Analysis

In the current study, a significance level of α = 0.05 was applied to control the risk of a type I error within a 5% threshold. For continuous variables, the mean (M) was selected as the central measure of tendency and standard deviation (SD) was used to represent the variance. For categorical data, the number of observations (*n*) and percentage distributions within categories were reported to summarize the frequency and proportion of each group. The reliability of the individual scales was assessed using Cronbach’s alpha to evaluate internal consistency [13]. Confidence intervals for Cronbach’s alpha were calculated using the Feldt method [14]. Questionnaire data, based on ordinal item responses scored across more than two categories, were analyzed using the rating scale model (RSM) (sometimes called the polytomous Rasch model), applying conditional maximum likelihood estimation (CMLE) [15]. An explanation of RSM notation and statistical measures is provided in Appendix A.

Residual-based model–data fit indicators, including unweighted (outfit) and weighted (infit) mean square error (MSE) statistics, were used to evaluate the numeric summaries of the residuals. Fit statistics were calculated for both individual items and persons to assess the quality of the measurement procedure and the alignment of the data with the underlying model assumptions.

The relationship between the latent variable and the probability of a particular response was represented by model-expected item response functions (IRFs), which were overlaid with empirical (observed) IRFs, often referred to as item characteristic curves (ICCs). Separate plots were generated for each item to visually examine the alignment of the observed data with model expectations. Furthermore, a person–item map was constructed to display the locations of item and threshold parameters alongside the distribution of person parameters along the latent continuum, providing a comprehensive view of the interaction between items and persons [16].

Rasch reliability of the separation statistic was calculated for each facet of the model, including items and persons, to estimate the extent to which the measurement procedure could distinguish between individuals and items on the latent continuum [17,18,19,20].

To complement the item-level analysis based on the Rasch model, additional exploratory and confirmatory factor analyses (EFA and CFA) were conducted to assess the construct validity of the Polish version of the KORQ. These analyses aimed to verify whether the underlying latent structure of the questionnaire aligns with the original two-subscale model (activity limitation and symptoms).

Exploratory factor analysis (EFA) was performed using maximum likelihood extraction with oblimin rotation, allowing for correlation between factors. The suitability of the data for factor analysis was first assessed to ensure sampling adequacy and inter-item correlation. The number of retained factors was based on multiple criteria, including eigenvalues, scree plot inspection, and theoretical interpretability. The factor structure was then evaluated to confirm whether items were grouped as expected into their respective dimensions.

Confirmatory factor analysis (CFA) was subsequently conducted using the diagonally weighted least squares (DWLS) estimator, which is appropriate for ordinal data derived from Likert-type response formats. Two models were tested: a unidimensional model including all items and a two-factor model reflecting the original subscale structure of the KORQ. Standard model fit indices were used to compare the models and assess the adequacy of the proposed factor structure. All model parameters were examined to determine whether the data supported the intended construct validity.

Regression analysis aimed to identify factors influencing keratoconus severity and visual outcomes using the total KORQ as the dependent variable. Independent variables included demographic factors: age at disease detection, age at the time of the study, sex, uncorrected visual acuity (VISsc), corrected visual acuity (VISsc), keratometry (Kmax), pachymetry (CCT), and the severity of keratoconus. Corrective measures such as glasses and contact lenses were also included. The data were prepared by excluding missing values and checking assumptions, including linearity, homoscedasticity, and multicollinearity, which were all satisfied.

### 2.3. Characteristics of the Statistical Tool

Analyses were conducted using the R statistical language (version 4.3.3) [21] on Windows 11 pro 64 bit (build 22631), using the mirt (version 1.43) [22], eRm (version 1.0.6) [23], report (version 0.5.8) [24], lattice (version 0.22.5) [25], get summary (version 1.7.2) [26], reshape2 (version 1.4.4) [27], ggplot2 (version 3.5.0) [28], dplyr (version 1.1.4) [29], psych (version 2.4.6.26) [30], kableExtra (version) [31], and knitr (version 1.46) [32] packages.

## 3. Results

### 3.1. Demographic and Clinical Characteristics of the Participants

The analysis included 100 adult patients with keratoconus. The baseline demographic and clinical characteristics of the study population, including sex distribution, age at the time of examination and disease detection, methods of visual correction, and key clinical parameters, such as visual acuity, keratometry, and corneal thickness, are reported in Table 1.

The findings in Table 1 suggest that keratoconus imposes a substantial burden on both the visual function and quality of life. Patients often face limitations in activities that require sharp visual acuity, such as reading, driving, or using digital devices. Furthermore, asymmetry in visual function can exacerbate difficulties in tasks requiring binocular coordination, such as sports or detailed manual work.

Specifically, the average age at disease detection (29.21 years) indicates that keratoconus typically manifests in early adulthood, aligning with its known progressive course during the second and third decades of life. By the time of examination (mean age 41.41 years), these patients represent a mix of progressive and stabilized cases, although the high prevalence of corneal abnormalities (presence of keratoconus in 84%) suggests that the majority of individuals are still experiencing notable disease-related challenges.

Best corrected visual acuity (VIScc) shows an asymmetry between worse and better eyes, with mean values of 0.31 and 0.65, respectively. While the better eye often retains relatively functional vision, the worse eye exhibits substantial visual impairment. This discrepancy contributes to binocular visual challenges, such as difficulty in depth perception and overall visual stability, which further influence daily activities. The mean difference in VIScc (0.33) highlights the variability in disease severity between eyes.

The mean Kmax value (49.21 D) in the better eye reflects advanced corneal steepening, which is consistent with keratoconus progression. Combined with a mean central corneal thickness (CCT) of 486.89 µm, which is markedly thinner than normal, these findings stress the cornea’s structural compromise. The average degree of cone severity (2.16) suggests that most patients fall within moderate disease stages, which are often associated with significant visual distortion and dependence on corrective measures.

The reliance on vision correction in this cohort is notable. Glasses (42%) and contact lenses (31%) are common, but a significant subset relies on advanced methods, such as rigid gas-permeable lenses (RGP lenses), including scleral or hybrid lenses (29%). These modalities are often necessary in patients with irregular astigmatism or advanced corneal steepening, where glasses or standard lenses fail to provide adequate correction. The 33% of patients without correction represent those who are unable or unwilling to tolerate contact lenses due to discomfort, cost, or limited access to specialized care. In this subgroup, uncorrected vision is likely to significantly impair daily functioning.

### 3.2. Eye-Specific Clinical Characteristics

Table 2 below presents the eye-specific clinical characteristics of the studied cohort, focusing on key parameters for both the right and left eyes. These include the presence or absence of keratoconus, prior interventions such as corneal transplantation or corneal crosslinking (CLX), and detailed ophthalmic measurements such as corrected visual acuity (VIScc), spherical equivalent (SE), keratometry (Kmin, Kmax), CCT (central corneal thickness, µm), and MCT (minimum corneal thickness, µm). These data provide a comprehensive comparison between the right and left eyes of the patients, highlighting potential asymmetries in disease presentation.

The results emphasize keratoconus’s asymmetric nature, with the study cohort’s left eye showing slightly worse structural and functional parameters. The observed asymmetries in corneal parameters and visual acuity suggest that the left eye contributes more to the overall disease burden and could influence patient-reported outcomes, including activity limitations and symptoms captured by instruments such as the KORQ.

Keratoconus was slightly more frequent in the left eye (94%) than in the right eye (84%), suggesting a tendency for greater disease progression or severity in one eye. This asymmetry is further reflected in the higher rate of corneal transplantation in the left eye (23%) than in the right eye (14%). Corneal crosslinking (CLX) was performed slightly more often in the right eye (13%) than in the left eye (10%), indicating earlier intervention in the right eye or differences in disease progression.

The corrected visual acuity (VIScc) was marginally worse in the left eye (mean 0.45) than in the right eye (mean 0.51), reflecting a slight functional disparity. This is consistent with other corneal parameters, such as keratometry and corneal thickness, which show subtle but clinically relevant differences. The mean spherical equivalent (SE) was more negative in the right eye (−3.73 D) than in the left eye (−3.15 D), indicating a greater degree of myopia or refractive error in the right eye. The minimum keratometry (Kmin) and maximum keratometry (Kmax) values were both slightly higher in the left eye (48.43 D and 52.47 D, respectively) than in the right eye (47.72 D and 51.45 D). These findings indicate that the left eye in the study cohort exhibited more pronounced corneal steepening, which is characteristic of keratoconus progression.

Astigmatism, represented by the difference between K2 and K1 (K2–K1), was slightly greater in the left eye (mean 4.07 D) than in the right eye (mean 3.73 D), further supporting the observation of greater corneal irregularity in the left eye. This is consistent with the degree of cone severity, which was also slightly higher in the left eye (mean 2.88) than in the right eye (mean 2.73). Corneal thickness measurements reinforced this asymmetry. The central corneal thickness (CCT) was thinner in the left eye (mean 472.44 µm) than in the right eye (mean 476.58 µm), and the minimum corneal thickness (MCT) followed a similar pattern, with the left eye (mean 431.51 µm) being slightly thinner than the right eye (mean 439.55 µm). These findings are consistent with keratoconus, where progressive thinning and steepening of the cornea occur, often asymmetrically between eyes.

### 3.3. Reliability Analysis of the Responses of the KORQ

The activity limitation scale of the KORQ (Q1–Q18) exhibited excellent internal consistency, as indicated by a Cronbach’s alpha of α_c_ = 0.95 (95% CI: 0.93–0.96). This high level of reliability was further supported by leave-one-out cross-validation, where alpha values consistently ranged between 0.94 and 0.95, demonstrating the scale’s robustness.

The responses to items Q1–Q11 on the symptoms scale of the KORQ demonstrated high reliability, with α_c_ = 0.87 (95% CI: 0.83–0.91). Furthermore, reliability remained stable under leave-one-out cross-validation, with Cronbach’s alpha consistently ranging from 0.85 to 0.87.

**Figure A1:**
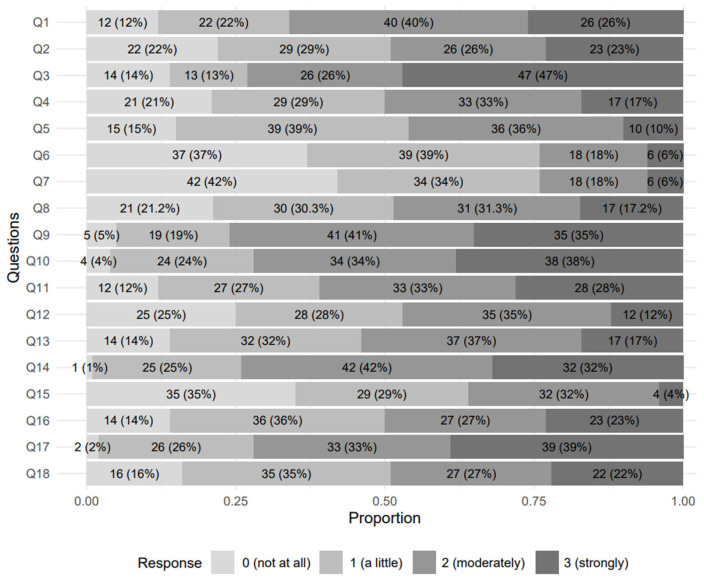
Distribution of responses for the activity limitation scale of the KORQ.

**Figure A2:**
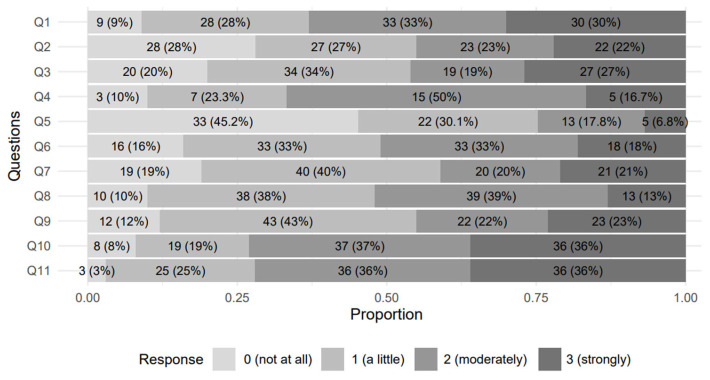
Distribution of responses for the symptoms scale of the KORQ.

The distribution of responses across the 18 questions of the activity limitation scale is presented in Figure A1 (Appendix A). Similarly, the distribution of responses for the 11 questions of the symptoms scale is illustrated in Figure A2 (Appendix A).

### 3.4. RSM Analysis for the Activity Limitation Scale of the KORQ

**Figure 1:**
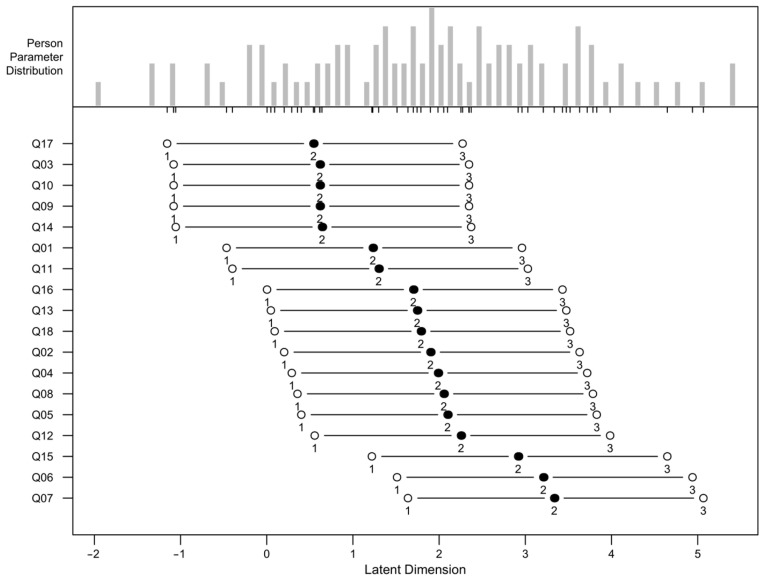
The calibration of the patients and items on the logit scale represents the latent variable ordered by the level of visual impairment for the KORQ activity limitation scale. Black dots are the central item difficulty, while white dots show the thresholds for category transitions. The numbers correspond to the items in the dataset, and their position on the latent dimension (*X* axis) indicates its difficulty level.

Figure 1 illustrates the calibration of patients and items on the logit scale, representing the latent variable associated with activity limitation. The calibrations correspond to the results reported in Table A2 and Table A3 (Appendix A) for items and patients, respectively. The horizontal axis, labeled the “Latent Dimension”, represents the logit scale, with lower values indicating less favorable attitudes toward activity limitation and higher values reflecting more favorable attitudes.

The central panel of the figure displays the item difficulty locations for the 18 activity limitation items analyzed. The *y*-axis contains item labels arranged in increasing order of vision impairment, as estimated by the RSM. Items representing lower levels of limitation are located at the top, whereas items reflecting higher levels of limitation are positioned at the bottom. For each item, a solid circle represents the overall location estimate. The solid circle is connected to two open circles, representing the locations of the rating scale category thresholds. The thresholds are labeled as follows: “1” denotes the transition between response categories x = 0 and x = 1, “2” indicates the transition between x = 1 and x = 2, and “3” signifies the transition between x = 2 and x = 3. These thresholds are detailed further in Table A4 (Appendix A).

Items located higher on the *y*-axis, such as Q17 (seeing small objects at a distance), Q3 (driving at night), and Q10 (interference from oncoming lights), represent more challenging tasks for patients, corresponding to greater perceived activity limitations. These items are likely to be particularly problematic for individuals with severe vision impairment and require higher levels of visual functioning to accomplish.

In contrast, items located lower on the *y*-axis, such as Q7 (avoiding objects on paths), Q6 (walking up/down steps), and Q15 (household tasks), are less difficult and represent activities that are generally less impacted by vision impairment. These items may reflect the baseline activities that even patients with substantial limitations can perform.

The patient parameter histogram in the upper panel reveals the distribution of the patients’ abilities in the latent dimension. The overlap between patient abilities and item difficulties suggests that the scale is well-targeted to the study population. However, the slight clustering of patients toward higher abilities indicates that some items may not adequately capture the full range of activity limitations, particularly in patients with minimal visual impairment. This suggests that adding more challenging items could enhance the scale’s ability to differentiate higher levels of visual function.

The calibration of patients’ difficulties at the patient and item level using average logit-scale calibrations, standard errors, and model–data fit statistics are summarized in Table A1 (Appendix A).

The mean logit-scale location for items is anchored at 0.00, aligning with the standard Rasch model assumption that item difficulties are centered around the mean of the latent trait. In contrast, the mean patient location is 1.87 logits, with higher standard deviation in patient logit locations (1.57 logits) than in items (0.89 logits), which highlights the greater variability in patient abilities. The standard error of measurement is slightly higher for patients (mean of 0.37 logits) than for items (mean of 0.16 logits), which is expected due to the inherent variability in patient responses. The small standard deviations of the standard errors (0.03 for items and 0.07 for patients) demonstrate consistency in the precision of these estimates, underscoring the reliability of the measurements.

Fit statistics indicate that the scale performs well in terms of model–data fit. Both items and patients demonstrate mean infit and outfit mean square error (MSE) values close to 1.00, indicating that the observed data align well with the expectations of the Rasch model. The standard deviations for both items and patients are within acceptable ranges, with slightly greater variability observed in patient fit statistics. This indicates that while most patients conform to the model’s expectations, a small subset may exhibit response patterns that deviate from the model, potentially due to unique functional limitations or measurement noise. The standardized fit statistics show means close to 0, further supporting the overall fit of the data to the model.

The separation reliability values are particularly noteworthy. The item separation reliability is 1.00, indicating excellent precision in distinguishing item difficulties on the latent scale. Similarly, the patient separation reliability of 0.94 reflects robust discrimination of patient abilities, suggesting that the scale is well-suited for differentiating between individuals with varying levels of activity limitation. These high-reliability values support the use of the activity limitation scale in both the clinical and research settings.

Table A2 (Appendix A) presents detailed results for the 18 items included in the analysis, where items are ordered by their overall logit-scale location (i.e., vision impairment) from less to more severe. For each item, the average rating is presented, followed by the overall logit-scale location (δ), SE, and model–data fit statistics. Similarly, Table A3 summarizes the results at the patient level. Appendix A contains a description of the terminology and the metrics used in the analysis of the rating scale model (RSM).

### 3.5. RSM Analysis with CMLE for the Symptoms Scale of the KORQ

**Figure 2:**
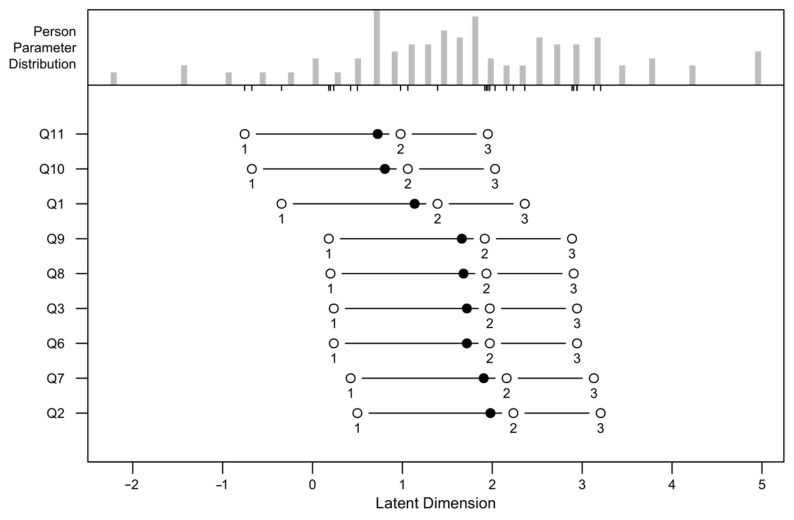
The calibration of patients and items on the logit scale represents the latent variable ordered by the level of visual impairment on the KORQ symptoms scale. Black dots are the central item difficulty, while white dots show the thresholds for category transitions. The numbers correspond to the items in the dataset, and their position on the latent dimension (*X* axis) indicates its difficulty level.

The calibration of patients and items on the logit scale in Figure 2 provides a detailed picture of how various symptoms of visual impairment affect individuals, as assessed by the KORQ symptoms scale, and summarizes the thresholds for each item in Table A8. (Appendix A). The items are ordered by their relative difficulty, with higher logit values indicating less frequent or less severe symptoms and lower logit values corresponding to more common or more severe symptoms of visual impairment.

According to Figure 2 together with Table A7 (Appendix A), item Q11, reflecting trouble in smoky environments, emerges as the most severe and impactful symptom, positioned at the far left of the scale. This suggests that smoke exposure significantly worsens visual discomfort, likely due to surface irritation and reduced visibility. For patients regularly exposed to such environments, this symptom can severely impair daily functioning, highlighting the need for protective measures, such as specialized eyewear or environmental avoidance strategies. Similarly, Q10, which relates to discomfort on dusty days, is highly impactful, exacerbating irritation and inflammation for individuals with preexisting ocular surface conditions, such as dry eye syndrome.

Item Q1, representing trouble with distorted vision, also ranks among the most severe symptoms on the scale. Distortion negatively affects core visual tasks, such as reading, navigating, and facial recognition, all of which require clarity and precision.

Items such as Q9, associated with discomfort on dry days, and Q8, reflecting trouble when tired, while positioned slightly further to the right, remain significant. Dry weather intensifies symptoms by aggravating ocular surface dryness, which is a common issue in many patients with visual impairment. Similarly, fatigue amplifies visual strain and often compounds other symptoms, creating a cascading effect that further impairs functionality.

Item Q3, which reflects interference from bright sunny days, and Q6, related to trouble with dry eyes, occupy mid-range positions on the severity scale. Glare from bright sunlight is a common complaint that limits outdoor activity and reduces visual comfort, reinforcing the need for interventions, such as polarized sunglasses or adaptive lenses. Dry eyes, however, remain one of the most prevalent contributors to discomfort and visual dysfunction.

Item Q7, which assesses trouble on windy days, is positioned further to the right and is less severe but still clinically relevant for individuals who experience discomfort due to wind exposure. Wind exacerbates dryness and irritation, particularly for those with already compromised ocular surfaces.

Q2, reflecting trouble with glare and the need to wear sunglasses all the time, is the least impactful symptom, positioned at the far right of the scale. While glare sensitivity remains a common issue, it appears to impose a lesser burden than more severe symptoms, such as smoky environments or distorted vision.

The omission of Q4 and Q5 due to substantial missing data (70% and 26%, respectively) may limit the completeness of the analysis and raise questions about the clarity or relevance of these items. While their exclusion reduces the scope of the findings, it may also reflect challenges in how these items are interpreted or applied by patients.

The patient-specific results presented in Table A7 provide additional findings on the variability and distribution of symptom severity as experienced by the respondents.

Based on Table A5 (Appendix A), separation reliability is extremely high for items (1.00), confirming excellent precision in distinguishing item difficulty, while patient reliability (0.86) is strong but reflects slightly more variability in symptom severity among patients. Standard errors for item locations are small (mean 0.14), emphasizing the precision of item calibration, although they are slightly higher for items at the extremes, such as Q11 (0.19) and Q9 (0.14), which represent more severe symptoms.

The fit statistics overall indicate good alignment with the Rasch model. The mean outfit and infit MSE values for both items and patients are close to the expected range of 1.00, with minimal misfit. However, Q1 (distorted vision) shows elevated outfit (1.31) and infit (1.30) MSE values, suggesting some unpredictability in responses. This discrepancy may reflect the complexity of the symptom, which could be interpreted differently by patients depending on their underlying conditions. Other items, such as Q9 and Q11, show a strong fit but are positioned at the severe end of the scale, indicating their relevance in addressing the most debilitating symptoms.

In summary, the conducted analysis demonstrated that the fit and functioning of most items suggest that the Polish version of the KORQ is a reliable and valid tool for assessing visual impairment-related symptoms. However, the analysis identified problematic questions within the survey, raising concerns about their interpretability and their contribution to the scale’s overall utility. Specifically, Q1 from the activity limitations subscale and Q4 and Q5 from the symptoms scale were identified as problematic.

Following a comprehensive analysis, a decision was made to exclude two of the problematic questions identified in the study (Q4 and Q5 on the symptoms scale). The rationale and detailed considerations underlying this decision are thoroughly documented in the Section 4.

### 3.6. Exploratory and Confirmatory Factor Analysis

#### 3.6.1. Exploratory Factor Analysis (EFA)

An exploratory factor analysis was conducted using maximum likelihood extraction with oblimin rotation. The data were suitable for factor analysis, as confirmed by the Kaiser–Meyer–Olkin (KMO) measure of sampling adequacy (KMO = 0.90) and Bartlett’s test of sphericity (χ^2^ = 1935.64, df = 351, *p* < 0.001). Two factors were extracted based on eigenvalues greater than 1 and scree plot analysis, corresponding to the original subscales of the KORQ: activity limitation and symptoms. These two factors explained a total of 53% of the variance (39% and 13%, respectively). The majority of items loaded clearly onto their respective factors. Items Q2 and Q3 (symptom subscale) demonstrated moderate cross-loadings but did not affect the overall interpretability of the factor structure. The item-level factor loadings are presented in Table A9 (Appendix A).

#### 3.6.2. Confirmatory Factor Analysis (CFA)

A confirmatory factor analysis was subsequently performed using the DWLS estimator. Two models were compared: a unidimensional model including all items and a two-factor model reflecting the original subscale structure. The two-factor model demonstrated superior fit (χ^2^(323) = 688.82, CFI = 0.981, TLI = 0.980, RMSEA = 0.104, SRMR = 0.109) to the one-factor model (χ^2^(324) = 924.53, CFI = 0.970, TLI = 0.967, RMSEA = 0.125, SRMR = 0.124). The chi-square difference test confirmed that the two-factor model fit the data significantly better (Δχ^2^ = 50.63, Δdf = 1, *p* < 0.001).

All the standardized factor loadings in the two-factor model were statistically significant (*p* < 0.001). The latent factors were moderately and significantly correlated (r = 0.729, *p* < 0.001).

### 3.7. Regression Analysis

After eliminating the problematic questions, a regression analysis was conducted. Regression analysis aimed to identify factors influencing keratoconus severity and visual outcomes, using the total KORQ score as the dependent variable. Regression analysis revealed significant predictors of keratoconus severity and visual outcomes. Age at disease detection was a marginally significant predictor (β = −0.02434, *p* = 0.061). Uncorrected visual acuity (VISsc) in the better eye was a significant predictor (β = −1.46793, *p* = 0.026). Similarly, corrected visual acuity in the better eye (VIScc) was significantly associated with the outcomes (β = 1.70527, *p* = 0.035), highlighting the importance of effective optical correction.

The severity of keratoconus also had a significant positive association with the total KORQ score (β = 0.51828, *p* = 0.023). Conversely, factors such as the corneal thickness parameters (MCT, β = −0.00200, *p* = 0.788; CCT, β = 0.00155, *p* = 0.834) and maximum corneal curvature in the better eye (Kmax, β = −0.00237, *p* = 0.946) did not show significant relationships with visual outcomes. Other demographic and clinical factors, including sex (β = 0.29685, *p* = 0.320), use of glasses (β = −0.22569, *p* = 0.261), and use of contact lenses (β = −0.53884, *p* = 0.377), did not significantly affect visual outcomes.

## 4. Discussion

### 4.1. Reliability and Construct Validity of the Polish KORQ 

The study was to adapt and assess the psychometric properties of the Polish version of the KORQ. The results indicate that this instrument is reliable and valid for measuring the quality of life of patients with keratoconus. The Rasch analysis showed good item fit, with all items demonstrating adequate fit statistics (infit and outfit mean square values close to 1), confirming unidimensionality within each of the two subscales: activity limitation and symptoms. The person separation reliability index was 0.94, and the item separation reliability index was 1.00, indicating excellent measurement precision and the ability of the scale to differentiate between patients with varying levels of quality of life.

Additionally, exploratory and confirmatory factor analyses (EFA and CFA) were conducted as part of the validation process to assess the theoretical two-factor structure of the KORQ. The EFA identified two distinct factors corresponding to the activity limitation and symptoms domains, together accounting for 53% of the total variance. Most items loaded clearly onto their respective factors, except for two items (Q2 and Q3 from the symptoms subscale), which exhibited moderate cross-loadings. These cross-loadings did not substantially affect the interpretability of the factor structure and likely reflect the natural interplay between the symptoms and functional limitations. Given the strong support for the original two-factor model in the CFA, no modifications to the questionnaire were deemed necessary.

The subsequent CFA supported the two-factor model as a significantly better fit than the one-factor solution, with all factor loadings being statistically significant. The two latent factors were positively correlated (r = 0.729, *p* < 0.001), indicating related but conceptually distinct constructs. While RMSEA (0.104) and SRMR (0.109) slightly exceeded conventional thresholds (<0.08), these values are considered acceptable in validation studies with modest sample sizes, especially when other fit indices, such as CFI (0.981) and TLI (0.980), indicate excellent model fit and the model is grounded in a strong theoretical framework [33,34].

The strong alignment observed in the Rasch analysis together with the confirmatory support for the theoretical structure from EFA and CFA, despite minor deviations such as cross-loadings and borderline fit indices, collectively strengthen the evidence for the construct validity of the Polish KORQ.

### 4.2. Discussion of the Demographic and Clinical Characteristics of Participants and the Methodology

The first part of the discussion focuses on the analysis of the demographic and clinical characteristics of the patients, referencing the results obtained during the third phase of the study conducted by Khadka et al., the authors of the questionnaire [6]. This phase was deliberately selected for comparison, as it provided compelling evidence supporting the validity and reliability of the final version of the KORQ, designed to accurately measure the impact of keratoconus on patients’ quality of life using advanced psychometric methods. Furthermore, the results from this phase represent the most refined and thoroughly validated version of the questionnaire, making it a robust benchmark for our analysis. The observed differences in methodology and the results of the regression analysis will also be discussed in detail.

In our study, 20% of participants were female and 80% male, which contrasts significantly with the Australian study, where 57.4% were female and 42.6% male. The observed gender disparity, with a higher proportion of women than men, likely reflects the characteristic demographics of our patient population. A similar trend was demonstrated in our previous study on keratoconus [35]. The average age at the time of disease detection in our study was 29.21 years (SD = 12.39), while the average age at the time of the examination was 41.41 years (SD = 14.91). In comparison, the mean age in the Australian phase 3 study was 45 years (SD = 14.7). Regarding vision correction methods, 42% of our participants used glasses, 29% used rigid gas-permeable lenses (including scleral and hybrid lenses), and 33% did not use any form of vision correction. In the Australian study, 33.7% of participants used glasses, 58% wore contact lenses (type not specified), and 7.7% did not use any correction. These results show a higher percentage of glasses users and individuals without correction in our study but fewer participants using contact lenses overall. Here, we will refer to the results from other European countries [7,8,36]. The data reveal notable regional disparities in the use of vision correction methods among patients with keratoconus. In Poland, 33% of patients do not utilize any form of vision correction, significantly higher than in Italy (13%), Germany (13.8%), and Denmark (20.5%). Contact lens usage is also comparatively low, with Poland reporting 29%, while Germany leads at 57.7% (35.4% rigid gas-permeable and 22.3% soft lenses), followed by Denmark at 39% and Italy at 17% (rigid gas-permeable) and 10% (soft lenses). These differences suggest systemic challenges in Poland, including the limited availability of specialized lenses, socioeconomic barriers, and potentially lower awareness among patients and practitioners regarding advanced correction options. The lack of data on visual acuity in the Australian phase 3 study makes it difficult to draw definitive conclusions or perform direct comparisons in this area.

For treatment methods, 37% of our participants had undergone corneal transplantation, 20% had received crosslinking (CLX), and 52% had not received either treatment. In the Australian study, 26% had undergone corneal transplantation, 18.3% had received crosslinking, and 50.3% had not received treatment. This indicates a higher percentage of corneal transplant recipients and a slightly higher percentage of crosslinking procedures in our study. There was a relatively high proportion of corneal transplants in our group; however, it is important to note that our inclusion criteria allowed the presence of a corneal transplant in one eye provided that keratoconus was present in the other eye. This ensures that all participants had at least one eye with active keratoconus, maintaining the relevance of their responses regarding vision correction methods.

In our study, we employed a slightly different methodology from the authors of the original version of the KORQ, who, in the third phase of their study, mailed the questionnaire to participants. In contrast, in our approach, the questionnaire was handed directly to patients during their visit to the Ophthalmology Clinic. This allowed patients to ask questions and seek clarification if any part of the questionnaire seemed unclear. Additionally, during the visit, the diagnosis was verified and the current severity of the disease was assessed. This method ensured more comprehensive and precise data collection by combining patients’ perspectives with their verified clinical condition.

Our regression analysis identified key predictors of keratoconus severity and visual outcomes, including uncorrected visual acuity (VISsc), corrected visual acuity (VIScc), and the severity of keratoconus, all of which significantly impacted the patient-reported quality of life measured by the KORQ scores. In contrast, corneal thickness (MCT, CCT), maximum corneal curvature (Kmax), demographic factors such as gender, and the use of corrective lenses were not significantly associated with visual outcomes. These findings emphasize the critical role of visual acuity and keratoconus severity in understanding the disease’s impact on patient’s lives. Notably, our results are consistent with those of the phase 3 KORQ study, which also highlighted the significance of visual acuity and keratoconus severity while finding no meaningful associations with corneal thickness, Kmax, or demographic factors. Despite differences in the study methodologies and demographic characteristics between the two studies, the regression results demonstrate remarkable similarity. This alignment strengthens the validity of both analyses and underscores the importance of visual acuity and patient-reported outcomes as primary indicators for assessing keratoconus and its treatment outcomes.

### 4.3. Problematic Items and Final Version of the Polish KORQ 

During the validation of the Polish version of the KORQ, three questions—Q1 from the activity limitation subscale and Q4 and Q5 from the symptoms subscale were identified as problematic. The discussion regarding these questions focused on their utility, validity, and impact on the overall quality of the tool. The key aspects of this analysis are presented below.

Question Q1 (activity limitation subscale) showed elevated infit (1.31) and outfit (1.30) values, suggesting that responses to this question may be less predictable within the Rasch model framework. The distribution of thresholds for Q1 (−0.34, 1.39, 2.36), however, is evenly spaced, indicating that the response categories are distinguishable. Given that Q1 addresses distorted vision, which is a key symptom of keratoconus and one of the main concerns reported by patients, we concluded that this question should be a part of the questionnaire despite the weaker statistical fit. At the same time, we reviewed the translation of the question and determined that the translation does not require any modifications.

For questions Q4 and Q5 (symptoms subscale), we recorded a significant percentage of missing data (70% and 26%, respectively). In consultation with statisticians, we decided to omit these questions from the Rasch analysis. It was not an easy decision, especially since in previously published studies, these questions have been handled differently, even though similar issues were observed to those in our study. For instance, researchers from Italy [8] reported that a high percentage of respondents selected “not applicable” for Q4 (52.8%) and Q5 (25.7%) from the symptoms subscale. Despite this, the researchers did not remove the questions from the analysis. Bak-Nielsen et al., in their study on the validation of the Danish version of the KORQ, noted that a high percentage of respondents selected “not applicable” (33.3%) for the question concerning the discomfort associated with wearing an RGP contact lens [36]. Danish researchers decided to remove this question from the questionnaire. We recognize that omitting the questions may limit the completeness of the analysis and reduce the scope of the findings; however, this decision was made to improve the quality and reliability of the tool, considering the significant percentage of missing data and potential interpretative challenges that could have affected the validity of the analysis. Q4 and Q5 were removed from the questionnaire due to their limited utility, resulting from a high number of missing responses and potential interpretation issues. The removal of these questions was necessary to improve the quality and reliability of the tool.

After the removal of the problematic questions, the final Polish version of the KORQ was created. This version reflects the results of the statistical analysis and adjustments made to enhance the tool’s reliability, validity, and cultural relevance. The finalized Polish KORQ maintains its ability to effectively assess the impact of keratoconus on patients’ quality of life, with improved clarity and consistency across its items. The Polish version of the KORQ is attached to this study (Supplementary Materials).

Our findings suggest removing or modifying these problematic questions to improve the Polish version of the KORQ. This approach is consistent with the recommendations of other researchers, who have emphasized the importance of the cultural adaptation of questionnaires to ensure their validity and reliability across different populations [37,38].

This study aligns with the findings of other research that emphasizes the importance of visual acuity for the quality of life of patients with keratoconus. For example, a study conducted by Jones-Jordan et al. demonstrated that visual acuity in the better-seeing eye has the most significant impact on the patient’s quality of life, regardless of the stage of keratoconus [39]. Similarly, longitudinal studies evaluating the quality of life of patients with keratoconus before and after treatment with refractive laser and crosslinking, or crosslinking alone, have shown improvements in both quality of life and visual acuity [40,41].

### 4.4. Study Limitations

This study has several limitations. First, the relatively small sample size may have particularly influenced the results of the confirmatory factor analysis (CFA), potentially limiting the accuracy of the model fit indices. Second, all patients were recruited from a single university clinic, reducing the generalizability of the findings. Third, the patient group lacked diversity, especially concerning patients with early-stage keratoconus and female participants. Additionally, longitudinal data tracking changes over time or following interventions such as corneal crosslinking were not included, which could provide further insights into the clinical utility of the KORQ. Despite these limitations, the convergence of our findings with the theoretical model and consistency with previous validations suggest that the Polish version of the KORQ is a reliable and valid instrument for assessing the quality of life in patients with keratoconus. In our opinion, it is reasonable to conduct further studies involving a larger and more diverse patient population to confirm the stability of the obtained results.

## 5. Conclusions

The Polish version of the Keratoconus Outcomes Research Questionnaire (KORQ) has shown good psychometric properties, confirming its reliability and construct validity. Rasch analysis demonstrated excellent item fit and separation reliability, while both exploratory and confirmatory factor analyses supported the theoretical two-factor structure. The convergence of results across Rasch and classical factor analyses provides robust evidence for the instrument’s construct validity.

The final Polish KORQ is a culturally adapted, reliable instrument suitable for clinical and research use in assessing vision-related quality of life in patients with keratoconus.

Regression analysis further confirmed its clinical utility by identifying key factors influencing disease severity and visual outcomes.

## Figures and Tables

**Table 1:** Demographic and overall clinical characteristics of the studied cohort.

Characteristic	*N*	Distribution
Sex, *n* (%)	100	
Female		20 (20.00%)
Male		80 (80.00%)
Age at the time of detection of the disease, M (SD)	100	29.21 (12.39)
Age at the time of the examination, M (SD)	100	41.41 (14.91)
Presence of keratoconus (OU), *n* (%)	100	84 (84.00%)
Method of vision correction, *n* (%)		
Glasses	100	42 (42.00%)
Lenses	100	31 (31.00%)
RGP lenses (including scleral and hybrid lenses)	100	29 (29.00%)
Soft contact lenses	100	2 (2.00%)
No correction	100	33 (33.00%)
VIScc (visual acuity, corrected), M (SD)		
Worse eye	100	0.31 (0.26)
Better eye	100	0.65 (0.31)
Difference	100	0.33 (0.26)
Kmax (maximum keratometry, better eye), M (SD)	100	49.21 (6.84)
CCT (central corneal thickness, better eye), M (SD)	100	486.89 (73.20)
Stage of keratoconus	84	2.16 (1.30)
Methods of treatment		
Corneal transplantation	100	37 (37%)
Crosslinking (CLX)	100	20 (20%)
None of the above	100	52 (52%)

**Table 2:** Eye-specific clinical characteristics of the studied cohort.

Characteristic	*N*	Eye	
		Right, *n*_1_ = 100	Left, *n*_2_ = 100
Presence of keratoconus	200	84 (84.00%)	94 (94.00%)
Corneal transplantation	200	14 (14.00%)	23 (23.00%)
Crosslinking (CLX)	200	13 (13.00%)	10 (10.00%)
VIScc (corrected visual acuity)	200	0.51 (0.31)	0.45 (0.35)
SE (spherical equivalent, D)	199	−3.73 (2.91)	−3.15 (3.21)
Kmin (minimum keratometry, D)	200	47.72 (7.62)	48.43 (9.34)
Kmax (maximum keratometry, D)	200	51.45 (8.74)	52.47 (10.00)
K2-K1 (astigmatism, D)	200	3.73 (2.78)	4.07 (3.29)
Stage of keratoconus	152	2.73 (0.93)	2.88 (1.06)
CCT (central corneal thickness, µm)	200	476.58 (75.65)	472.44 (75.69)
MCT (minimum corneal thickness, µm)	200	439.55 (93.22)	431.51 (94.34)

## Data Availability

Data are available on request due to restrictions (e.g., privacy or ethical).

## Supplementary Materials

The following supporting information can be downloaded at: https://www.mdpi.com/article/10.3390/jcm14092959/s1, Validated Polish version of the KORQ.

## Appendix A

**Table A1:** Summary of the results from the analysis of the activity limitation scale of the KORQ.

Statistic	Items	Patients
Logit-scale location mean	0.00	1.87
Logit-scale location SD	0.89	1.57
Standard error mean	0.16	0.37
Standard error SD	0.03	0.07
Outfit MSE mean	0.96	0.96
Outfit MSE SD	0.24	0.52
Infit MSE mean	0.96	0.94
Infit MSE SD	0.22	0.48
Std. outfit mean	−0.32	−0.22
Std. outfit SD	1.48	1.44
Std. infit mean	−0.36	−0.27
Std. infit SD	1.56	1.43
Separation reliability	1.00	0.94

**Table A2:** The overall calibrations of individual items for the activity limitation scale of the KORQ.

Item	Average Rating	Item Location	Item SE	Outfit MSE	Std. Outfit	Infit MSE	Std. Infit
Q07	1.88	1.64	0.15	0.69	−1.84	0.77	−1.66
Q06	1.93	1.51	0.16	0.69	−1.92	0.75	−1.85
Q15	2.05	1.22	0.14	0.71	−1.95	0.73	−2.07
Q12	2.34	0.56	0.14	0.98	−0.07	1.01	0.09
Q05	2.41	0.40	0.16	0.65	−2.80	0.65	−2.90
Q08	2.43	0.36	0.16	1.26	1.75	1.33	2.22
Q04	2.46	0.29	0.15	0.92	−0.57	0.92	−0.55
Q02	2.50	0.20	0.16	1.16	1.11	1.16	1.15
Q18	2.55	0.09	0.14	0.89	−0.77	0.91	−0.62
Q13	2.57	0.05	0.16	1.00	0.06	1.06	0.47
Q16	2.59	0.00	0.16	1.06	0.44	1.08	0.65
Q11	2.77	−0.40	0.15	0.77	−1.65	0.80	−1.50
Q01	2.80	−0.47	0.14	1.06	0.44	1.07	0.56
Q14	3.05	−1.06	0.16	0.79	−1.34	0.75	−1.91
Q09	3.06	−1.08	0.16	0.97	−0.11	0.76	−1.82
Q10	3.06	−1.08	0.15	1.01	0.13	1.03	0.23
Q03	3.06	−1.08	0.14	1.62	3.21	1.44	2.77
Q17	3.09	−1.16	0.14	1.00	0.04	1.02	0.20

**Table A3:** The overall calibrations of individual patients for the activity limitation scale of the KORQ.

Patient id	Average Response	Person Location	Person SE	Outfit MSE	Std. Outfit	Infit MSE	Std. Infit
P1	0.33	−1.09	0.47	0.62	−0.72	0.69	−0.73
P2	2.22	3.19	0.36	1.80	2.11	1.64	1.78
P3	2.00	2.69	0.34	0.35	−2.77	0.32	−2.96
P4	1.39	1.49	0.33	1.74	2.11	1.79	2.22
P6	2.61	4.31	0.45	0.76	−0.45	0.58	−1.17
P7	2.39	3.61	0.39	1.38	1.09	1.48	1.37
P8	2.17	3.06	0.36	0.72	−0.89	0.74	−0.80
P9	2.33	3.46	0.38	0.75	−0.71	0.71	−0.87
P10	1.67	2.02	0.33	1.29	0.99	1.30	1.01
P11	1.50	1.70	0.33	1.88	2.41	1.94	2.55
P12	1.72	2.13	0.33	1.33	1.08	1.30	1.01
P13	1.61	1.91	0.33	0.36	−2.73	0.36	−2.73
P14	1.94	2.58	0.34	0.81	−0.55	0.81	−0.55
P15	2.67	4.52	0.48	0.66	−0.64	0.75	−0.56
P16	1.78	2.24	0.33	1.23	0.82	1.28	0.95
P17	1.61	1.91	0.33	1.13	0.50	1.15	0.58
P18	1.61	1.91	0.33	0.45	−2.21	0.46	−2.15
P19	0.78	0.22	0.36	0.99	0.07	1.04	0.24
P20	1.78	2.24	0.33	1.05	0.26	0.98	0.05
P21	2.83	5.40	0.63	1.80	1.12	1.20	0.52
P22	1.67	2.02	0.33	0.92	−0.17	0.89	−0.27
P23	2.56	4.11	0.43	0.45	−1.56	0.43	−1.86
P24	1.72	2.13	0.33	1.16	0.60	1.16	0.59
P25	1.11	0.94	0.34	1.56	1.66	1.65	1.86
P26	0.61	−0.20	0.39	0.54	−1.42	0.54	−1.55
P27	2.44	3.77	0.40	0.56	−1.32	0.47	−1.81
P28	2.00	2.69	0.34	0.46	−2.14	0.44	−2.21
P29	2.44	3.77	0.40	0.49	−1.60	0.55	−1.45
P30	2.39	3.61	0.39	0.46	−1.85	0.49	−1.78
P31	0.61	−0.20	0.39	0.78	−0.54	0.82	−0.45
P32	1.56	1.81	0.33	0.93	−0.13	0.94	−0.10
P33	1.06	0.82	0.34	0.67	−1.11	0.68	−1.04
P34	2.17	3.06	0.36	2.84	4.03	2.55	3.57
P35	1.39	1.49	0.33	0.96	−0.04	0.96	−0.02
P36	1.33	1.38	0.33	1.35	1.13	1.30	0.99
P37	1.06	0.82	0.34	0.92	−0.15	0.94	−0.09
P38	0.89	0.47	0.35	0.69	−0.98	0.63	−1.26
P39	0.28	−1.33	0.51	0.47	−1.01	0.56	−1.09
P40	0.72	0.08	0.37	1.08	0.34	1.03	0.21
P41	1.33	1.38	0.33	0.63	−1.31	0.63	−1.29
P42	1.89	2.46	0.34	0.59	−1.49	0.59	−1.45
P43	0.67	−0.05	0.38	0.68	−0.92	0.72	−0.84
P44	1.83	2.35	0.33	1.34	1.10	1.33	1.07
P45	0.44	−0.69	0.43	0.70	−0.64	0.78	−0.53
P46	1.61	1.91	0.33	0.52	−1.83	0.51	−1.89
P47	2.00	2.69	0.34	1.06	0.31	1.10	0.41
P48	1.94	2.58	0.34	2.14	2.94	2.21	3.05
P49	0.67	−0.05	0.38	0.77	−0.60	0.87	−0.29
P50	2.33	3.46	0.38	0.57	−1.39	0.53	−1.61
P51	0.67	−0.05	0.38	0.57	−1.34	0.54	−1.58
P52	1.89	2.46	0.34	1.14	0.53	1.08	0.35
P53	0.50	−0.52	0.41	0.80	−0.41	0.98	0.05
P54	1.33	1.38	0.33	0.46	−2.13	0.47	−2.10
P55	2.06	2.81	0.35	0.95	−0.07	0.97	0.00
P56	1.33	1.38	0.33	1.72	2.06	1.76	2.14
P57	2.56	4.11	0.43	1.73	1.60	1.31	0.89
P58	1.28	1.27	0.33	0.44	−2.24	0.44	−2.25
P59	1.11	0.94	0.34	0.51	−1.85	0.50	−1.92
P60	2.50	3.93	0.41	1.19	0.59	0.94	−0.06
P61	1.89	2.46	0.34	0.90	−0.22	0.94	−0.09
P62	1.00	0.71	0.34	1.20	0.70	1.26	0.88
P63	2.06	2.81	0.35	1.62	1.78	1.75	2.07
P64	2.39	3.61	0.39	1.18	0.60	1.33	0.99
P65	0.28	−1.33	0.51	0.58	−0.71	0.68	−0.70
P66	2.06	2.81	0.35	0.50	−1.87	0.47	−2.05
P67	0.61	−0.20	0.39	0.72	−0.73	0.82	−0.45
P68	1.28	1.27	0.33	0.99	0.07	1.00	0.09
P69	2.78	5.05	0.56	0.34	−1.27	0.46	−1.29
P70	1.11	0.94	0.34	0.58	−1.51	0.56	−1.59
P71	1.61	1.91	0.33	1.73	2.08	1.73	2.09
P72	2.83	5.40	0.63	1.87	1.19	1.23	0.58
P73	1.89	2.46	0.34	2.38	3.41	2.26	3.15
P74	1.22	1.16	0.33	0.61	−1.39	0.60	−1.44
P75	1.67	2.02	0.33	0.96	−0.03	0.96	−0.03
P76	1.72	2.13	0.33	0.38	−2.62	0.40	−2.51
P77	0.33	−1.09	0.47	1.03	0.22	0.95	0.00
P78	1.44	1.59	0.33	0.65	−1.22	0.63	−1.29
P79	0.83	0.34	0.36	0.49	−1.84	0.51	−1.82
P80	1.06	0.82	0.34	0.69	−1.01	0.68	−1.05
P81	1.44	1.59	0.33	1.16	0.61	1.19	0.70
P82	1.50	1.70	0.33	0.83	−0.49	0.83	−0.51
P83	1.50	1.70	0.33	0.81	−0.56	0.81	−0.58
P84	2.22	3.19	0.36	0.87	−0.30	0.86	−0.36
P85	1.00	0.71	0.34	1.28	0.92	1.19	0.68
P86	1.72	2.13	0.33	0.99	0.05	1.01	0.13
P87	0.94	0.59	0.35	1.35	1.10	1.42	1.30
P88	1.50	1.70	0.33	0.48	−2.03	0.47	−2.08
P89	0.17	−1.96	0.62	0.40	−0.81	0.67	−0.53
P90	2.11	2.93	0.35	1.32	1.03	1.23	0.77
P91	2.11	2.93	0.35	0.88	−0.29	0.86	−0.35
P92	0.44	−0.69	0.43	0.63	−0.87	0.75	−0.65
P93	1.28	1.27	0.33	0.52	−1.82	0.50	−1.90
P94	2.17	3.06	0.36	2.84	4.03	2.55	3.57
P95	2.72	4.77	0.51	0.49	−0.99	0.53	−1.18
P96	2.39	3.61	0.39	0.84	−0.37	0.73	−0.77
P97	0.94	0.59	0.35	0.97	0.01	0.94	−0.09
P98	2.44	3.77	0.40	0.48	−1.64	0.49	−1.71
P99	1.56	1.81	0.33	0.56	−1.65	0.54	−1.74
P100	0.78	0.22	0.36	0.72	−0.83	0.58	−1.45

**Table A4:** Estimated thresholds (non-scaled) for individual items on the activity limitation scale of the KORQ.

Item	Threshold 1	Threshold 2	Threshold 3
Q01	−0.47	1.23	2.96
Q02	0.20	1.90	3.63
Q03	−1.08	0.62	2.35
Q04	0.29	1.99	3.72
Q05	0.40	2.10	3.83
Q06	1.51	3.21	4.94
Q07	1.64	3.33	5.07
Q08	0.36	2.05	3.78
Q09	−1.08	0.62	2.35
Q10	−1.08	0.62	2.35
Q11	−0.40	1.30	3.03
Q12	0.56	2.25	3.98
Q13	0.05	1.74	3.48
Q14	−1.06	0.64	2.37
Q15	1.22	2.92	4.65
Q16	0.00	1.70	3.43
Q17	−1.16	0.54	2.27
Q18	0.09	1.79	3.52

**Table A5:** Summary of the results from the analysis of the symptoms scale of the KORQ.

Statistic	Items	Patients
Logit-scale location mean	0.00	1.80
Logit-scale location SD	0.47	1.37
Standard error mean	0.14	0.50
Standard error SD	0.02	0.14
Outfit MSE mean	0.93	0.93
Outfit MSE SD	0.17	0.55
Infit MSE mean	0.91	0.90
Infit MSE SD	0.18	0.52
Std. outfit mean	−0.49	−0.27
Std. outfit SD	1.11	1.38
Std. infit mean	−0.72	−0.31
Std. infit SD	1.38	1.38
Separation reliability	1.00	0.86

**Table A6:** The overall calibrations of individual items for the symptoms scale of the KORQ.

Item	Average Rating	Item Location	Item SE	Outfit MSE	Std. Outfit	Infit MSE	Std. Infit
Q2	2.39	0.50	0.13	0.95	−0.32	1.03	0.28
Q7	2.43	0.42	0.13	0.96	−0.25	0.81	−1.43
Q6	2.53	0.24	0.13	0.91	−0.54	0.96	−0.27
Q3	2.53	0.24	0.13	0.92	−0.52	0.93	−0.52
Q8	2.55	0.20	0.13	0.86	−0.92	0.80	−1.54
Q9	2.56	0.18	0.14	0.66	−2.55	0.71	−2.32
Q1	2.84	−0.34	0.13	1.31	1.81	1.30	2.08
Q10	3.01	−0.68	0.14	0.87	−0.70	0.93	−0.49
Q11	3.05	−0.76	0.19	0.91	−0.46	0.72	−2.25

**Table A7:** The overall calibrations of individual patients for the symptoms scale of the KORQ.

Patient id	Average Response	Person Location	Person SE	Outfit MSE	Std. Outfit	Infit MSE	Std. Infit
P1	1.78	1.98	0.42	2.48	2.79	2.53	2.87
P2	0.44	−0.55	0.58	0.69	−0.51	0.68	−0.53
P3	0.22	−1.43	0.77	1.36	0.69	1.12	0.40
P4	0.22	−1.43	0.77	0.96	0.18	0.96	0.16
P5	0.11	−2.21	1.04	1.59	0.83	1.09	0.40
P6	0.33	−0.93	0.65	0.62	−0.58	0.61	−0.63
P7	1.78	1.98	0.42	1.99	2.07	2.03	2.14
P8	0.67	0.03	0.51	0.98	0.11	0.87	−0.12
P9	1.44	1.46	0.42	1.59	1.35	1.57	1.31
P10	0.89	0.51	0.47	0.73	−0.50	0.73	−0.49
P11	1.00	0.72	0.45	0.85	−0.20	0.83	−0.24
P12	1.67	1.81	0.42	0.42	−1.73	0.42	−1.75
P13	1.22	1.10	0.43	0.15	−3.11	0.16	−3.01
P14	0.67	0.03	0.51	0.87	−0.15	0.93	0.00
P15	1.00	0.72	0.45	0.94	0.02	0.99	0.12
P16	1.00	0.72	0.45	0.11	−3.33	0.12	−3.26
P17	1.00	0.72	0.45	0.43	−1.48	0.43	−1.51
P18	1.22	1.10	0.43	0.86	−0.19	0.87	−0.16
P19	1.33	1.29	0.42	0.64	−0.82	0.66	−0.76
P20	1.56	1.64	0.42	2.28	2.45	2.30	2.48
P21	1.44	1.46	0.42	0.47	−1.47	0.45	−1.54
P22	1.22	1.10	0.43	1.38	0.93	1.41	0.98
P23	0.89	0.51	0.47	0.70	−0.58	0.74	−0.48
P24	1.00	0.72	0.45	0.69	−0.62	0.66	−0.71
P25	1.44	1.46	0.42	0.37	−1.89	0.39	−1.82
P26	1.33	1.29	0.42	0.85	−0.22	0.81	−0.33
P27	1.00	0.72	0.45	0.67	−0.68	0.66	−0.71
P28	1.00	0.72	0.45	0.11	−3.33	0.12	−3.26
P29	1.56	1.64	0.42	1.12	0.41	1.10	0.38
P30	1.44	1.46	0.42	0.40	−1.74	0.41	−1.70
P31	1.67	1.81	0.42	0.39	−1.87	0.40	−1.84
P32	2.11	2.52	0.44	0.86	−0.21	0.91	−0.09
P33	2.00	2.34	0.43	2.30	2.53	2.06	2.19
P34	1.11	0.91	0.44	0.12	−3.27	0.14	−3.16
P35	1.00	0.72	0.45	0.48	−1.30	0.47	−1.33
P36	0.78	0.28	0.48	0.81	−0.28	0.73	−0.48
P37	1.11	0.91	0.44	1.16	0.48	1.17	0.51
P38	1.33	1.29	0.42	0.58	−1.02	0.58	−1.03
P39	1.44	1.46	0.42	0.58	−1.07	0.60	−1.00
P40	1.67	1.81	0.42	1.60	1.39	1.57	1.35
P41	2.56	3.45	0.55	1.44	0.88	1.66	1.24
P42	2.44	3.17	0.50	1.20	0.55	1.18	0.52
P43	0.89	0.51	0.47	1.12	0.40	1.12	0.41
P44	1.00	0.72	0.45	0.38	−1.68	0.39	−1.64
P45	0.56	−0.24	0.54	1.10	0.36	1.14	0.45
P46	1.11	0.91	0.44	0.12	−3.27	0.14	−3.16
P47	1.67	1.81	0.42	0.98	0.08	0.99	0.11
P48	1.22	1.10	0.43	0.34	−1.93	0.33	−1.98
P49	2.11	2.52	0.44	1.82	1.75	1.71	1.60
P50	2.33	2.94	0.47	0.32	−1.92	0.29	−2.16
P51	1.89	2.16	0.42	0.40	−1.84	0.40	−1.85
P52	2.11	2.52	0.44	1.14	0.46	1.21	0.63
P53	2.33	2.94	0.47	2.26	2.19	2.00	1.94
P54	2.44	3.17	0.50	1.89	1.59	1.36	0.84
P55	1.67	1.81	0.42	1.08	0.32	1.06	0.28
P56	2.33	2.94	0.47	0.63	−0.79	0.54	−1.14
P57	2.33	2.94	0.47	0.53	−1.09	0.55	−1.09
P58	2.22	2.72	0.45	0.81	−0.32	0.86	−0.22
P59	2.44	3.17	0.50	0.36	−1.55	0.37	−1.65
P60	2.11	2.52	0.44	1.34	0.88	1.43	1.07
P61	2.22	2.72	0.45	1.03	0.22	1.08	0.32
P62	1.56	1.64	0.42	1.62	1.41	1.61	1.39
P63	1.00	0.72	0.45	0.92	−0.03	0.87	−0.15
P64	2.89	4.96	1.02	0.81	0.19	0.91	0.25
P65	1.33	1.29	0.42	0.50	−1.30	0.52	−1.25
P66	1.67	1.81	0.42	0.38	−1.91	0.39	−1.88
P67	0.67	0.03	0.51	0.96	0.08	0.87	−0.13
P68	1.44	1.46	0.42	0.16	−3.14	0.16	−3.14
P69	2.44	3.17	0.50	0.75	−0.39	0.66	−0.66
P70	1.11	0.91	0.44	1.51	1.15	1.52	1.17
P71	1.22	1.10	0.43	0.71	−0.59	0.71	−0.62
P72	1.67	1.81	0.42	1.00	0.13	0.98	0.09
P73	2.22	2.72	0.45	1.43	1.03	1.44	1.08
P74	1.56	1.64	0.42	1.74	1.62	1.75	1.64
P75	1.33	1.29	0.42	0.54	−1.16	0.52	−1.23
P76	2.67	3.78	0.61	0.77	−0.16	0.76	−0.24
P77	2.33	2.94	0.47	0.67	−0.65	0.61	−0.91
P78	1.67	1.81	0.42	1.01	0.15	1.02	0.18
P79	2.56	3.45	0.55	0.81	−0.17	0.75	−0.36
P80	2.67	3.78	0.61	0.84	−0.05	0.82	−0.11
P81	1.67	1.81	0.42	0.49	−1.46	0.49	−1.46
P82	2.89	4.96	1.02	0.81	0.19	0.91	0.25
P83	2.44	3.17	0.50	0.91	−0.02	0.80	−0.30
P84	2.11	2.52	0.44	0.16	−3.23	0.12	−3.64
P85	2.44	3.17	0.50	0.45	−1.24	0.43	−1.41
P86	1.89	2.16	0.42	0.43	−1.71	0.41	−1.80
P87	1.78	1.98	0.42	0.42	−1.76	0.42	−1.74
P88	1.56	1.64	0.42	1.27	0.74	1.27	0.75
P89	2.89	4.96	1.02	2.19	1.17	1.06	0.40
P90	2.78	4.23	0.73	0.60	−0.33	0.72	−0.18
P91	2.22	2.72	0.45	2.11	2.11	2.34	2.50
P92	1.44	1.46	0.42	1.10	0.38	1.11	0.39
P94	2.00	2.34	0.43	1.32	0.86	1.36	0.95
P95	2.67	3.78	0.61	0.76	−0.18	0.75	−0.26
P96	2.89	4.96	1.02	0.63	0.00	0.85	0.19
P97	2.22	2.72	0.45	0.85	−0.23	0.84	−0.28
P98	2.78	4.23	0.73	0.92	0.16	0.83	0.01
P99	1.56	1.64	0.42	1.80	1.71	1.78	1.68
P100	2.11	2.52	0.44	1.04	0.22	1.01	0.15

**Table A8:** Total variance explained by the extracted factors.

Item	Threshold 1	Threshold 2	Threshold 3
Q1	−0.34	1.39	2.36
Q2	0.50	2.23	3.21
Q3	0.24	1.97	2.94
Q6	0.24	1.97	2.94
Q7	0.42	2.16	3.13
Q8	0.20	1.93	2.91
Q9	0.18	1.92	2.89
Q10	−0.68	1.06	2.03
Q11	−0.76	0.98	1.95

**Table A9:** Exploratory factor loadings by item.

Item	Factor 1 (Activity Limitation)	Factor 2 (Symptoms)
Q1	0.59	-
Q2	0.76	-
Q3	0.67	-
Q4	0.86	-
Q5	0.76	-
Q6	0.75	-
Q7	0.76	-
Q8	0.55	-
Q9	0.75	-
Q10	0.61	-
Q11	0.71	-
Q12	0.61	
Q13	0.64	
Q14	0.73	
Q15	0.71	
Q16	0.75	
Q17	0.73	
Q18	0.74	
Q1	0.76	
Q2	0.49	0.36
Q3	0.56	0.37
Q6	-	0.7
Q7	-	0.74
Q8	-	0.47
Q9	-	0.9
Q10	-	0.67
Q11	-	0.52

RSM notation

Item (Person) Location: This is an item’s (patient’s) difficulty on the logit scale, calculated as the midpoint between its rating scale thresholds.

Item (Person) Standard Error (Item SE): This reflects the precision of the item (patient) location estimate.

Outfit Mean Square Error (Outfit MSE): This statistic measures the degree of unexpected responses, focusing on outliers. It is more sensitive to extreme responses that deviate from the expected model.

Standardized Outfit (Std. Outfit): This transforms the outfit MSE into a standard normal distribution to aid interpretation.

Infit Mean Square Error (Infit MSE): This measure is more sensitive to responses that are close to the item’s difficulty level, making it useful for detecting patterns in the middle of the scale.

Standardized Infit (Std. Infit): Similar to Std. Outfit, this standardizes the infit MSE, allowing easier identification of potential misfit.

Average Rating: This represents the mean response category chosen by patients for each item.
