# Supplementary material for: Validation of the Polish Version of the Keratoconus Outcomes Research Questionnaire: Tool for Vision-Related Quality of Life in Patients with Keratoconus
Source: J Clin Med. 2025 Apr 24;14(9):2959. doi: 10.3390/jcm14092959 (PMC12072925; doi:10.3390/jcm14092959)
Supplement: Supplementary file 1 [file jcm-14-02959-s001.zip › jcm-3517125-supplementary.pdf]

Keratoconus Outcomes Research Questionnaire (KORQ)

**DANE DEMOGRAFICZNE – uzupełnij lub zaznacz poprawną odpowiedź**			
**Pani / Pan**		**Data:**	
**Nazwisko:**			
**Imię:**			
**Adres:**			
**Kod Pocztowy:**			
**Numer telefonu:**<br>Stacjonarny:	Komórkowy:		Służbowy:
**Data urodzenia:**		**Płeć:** Kobieta / Mężczyzna	
**Czy zdiagnozowano u Pani/Pana stożek rogówki?**		Tak	Nie
**Wiek w momencie diagnozy**	……..…… lat	Opcjonalnie proszę podać rok wykrycia …..……... r.	
**Stożek rogówki dotyczy:**	Oka prawego	Oka lewego	Obojga oczu
**Korzystam z:**<br>Okularów	Miękkich soczewek kontaktowych	Twardych soczewek kontaktowych (RGP)	Żadne z wymienionych
**Jakie leczenie stożka rogówki zastosowano?**<br>Crosslinking<br>**Inne (proszę opisać)…………………………………**	Przeszczep rogówki	Żadne z wymienionych	
**Ostrość wzroku w okularach/soczewkach kontaktowych (Jeśli jest znana)**<br>Oko prawe:		Oko lewe:	
**Aktualna recepta na okulary lub soczewki kontaktowe (Jeśli jest znana)**<br>Oko prawe:		Oko lewe:	
**Inne choroby oczu (Jeśli są znane)**			

Kwestionariusz KORQ - wersja polskojęzyczna

Keratoconus Outcomes Research Questionnaire (KORQ)

PODSKALA OGRANICZEŃ W AKTYWNOŚCIACH

Proszę o zaznaczenie odpowiedzi, która wskazuje na to, jak Pani/Pana wzrok wpływa na zdolność do wykonywania poszczególnych czynności.

1. Jak bardzo Twój wzrok przeszkadza Ci w korzystaniu z ekranu komputera?

Nie dotyczy

Wcale Mało Umiarkowanie Mocno

2. Jak bardzo Twój wzrok przeszkadza Ci w prowadzeniu pojazdu w ciągu dnia?

Nie dotyczy

Wcale Mało Umiarkowanie Mocno

3. Jak bardzo Twój wzrok przeszkadza Ci w prowadzeniu pojazdu w nocy?

Nie dotyczy

Wcale Mało Umiarkowanie Mocno

4. Jak bardzo Twój wzrok przeszkadza Ci w rozpoznawaniu znaków drogowych?

Nie dotyczy

Wcale Mało Umiarkowanie Mocno

5. Jak bardzo Twój wzrok przeszkadza Ci w oglądaniu telewizji?

Nie dotyczy

Wcale Mało Umiarkowanie Mocno

6. Jak bardzo Twój wzrok przeszkadza Ci podczas wchodzenia/schodzenia ze schodów?

Nie dotyczy

Wcale Mało Umiarkowanie Mocno

7. Jak bardzo Twój wzrok przeszkadza Ci w omijaniu przedmiotów znajdujących się na Twojej drodze?

Nie dotyczy

Wcale Mało Umiarkowanie Mocno

8. Jak bardzo Twój wzrok przeszkadza Ci w wykonywaniu Twojej pracy?

Nie dotyczy

Wcale Mało Umiarkowanie Mocno

9. Jak bardzo Twój wzrok przeszkadza Ci w widzeniu z daleka?

Nie dotyczy

Wcale Mało Umiarkowanie Mocno

10. Jak bardzo oświetlenie padające w Twoją stronę utrudnia Ci zdolność widzenia i wykonywania czynności?

Nie dotyczy

Wcale Mało Umiarkowanie Mocno

11. Jak bardzo Twój wzrok przeszkadza Ci w wykonywaniu precyzyjnych czynności z bliska?

Nie dotyczy

Wcale Mało Umiarkowanie Mocno

12. Jak bardzo Twój wzrok przeszkadza Ci w uprawianiu hobby?

Nie dotyczy

Wcale Mało Umiarkowanie Mocno

13. Jak bardzo Twój wzrok przeszkadza Ci w rozpoznawaniu twarzy?

Nie dotyczy

Wcale Mało Umiarkowanie Mocno

14. Jak bardzo Twój wzrok przeszkadza Ci w widzeniu przy słabym oświetleniu?

Nie dotyczy

Wcale Mało Umiarkowanie Mocno

15. Jak bardzo Twój wzrok przeszkadza Ci w wykonywaniu prac domowych? (np. sprzątanie, zmywanie, prasowanie)

Nie dotyczy

Wcale Mało Umiarkowanie Mocno

16. Jak bardzo Twój wzrok przeszkadza Ci w ocenie odległości?

Nie dotyczy

Wcale Mało Umiarkowanie Mocno

17. Jak bardzo Twój wzrok przeszkadza Ci w widzeniu małych obiektów w oddali? (np. piłka golfowa, rzutki)

Nie dotyczy

Wcale Mało Umiarkowanie Mocno

18. Jak bardzo Twój wzrok przeszkadza Ci w wykonywaniu zadań związanych z widzeniem? (np. obsługa aparatu fotograficznego, mikroskopu, lornetki, itp.)

Nie dotyczy

Wcale Mało Umiarkowanie Mocno

PODSKALA OBJAWÓW WZROKOWYCH

1. Jak bardzo przeszkadza Ci zniekształcone widzenie?

Nie dotyczy

Wcale Mało Umiarkowanie Mocno

2. Jak bardzo przeszkadzają Ci oślepiające światła i nieustanne noszenie okularów przeciwsłonecznych?

Nie dotyczy

Wcale Mało Umiarkowanie Mocno

3. Jak bardzo jasne, słoneczne dni utrudniają Ci widzenie i wykonywanie codziennych czynności?

Nie dotyczy

Wcale Mało Umiarkowanie Mocno

4. Jak bardzo przeszkadza Ci suchość oczu?

Nie dotyczy

Wcale Mało Umiarkowanie Mocno

5. W odniesieniu do Twoich oczu i wzroku: Jak bardzo dokuczają Ci wietrzne dni?

Nie dotyczy

Wcale Mało Umiarkowanie Mocno

6. W odniesieniu do Twoich oczu i wzroku: Jak bardzo zmęczenie wpływa na Twoją zdolność widzenia?

Nie dotyczy

Wcale Mało Umiarkowanie Mocno

7. W odniesieniu do Twoich oczu i wzroku: Jak bardzo dokucza Ci suche powietrze?

Nie dotyczy

Wcale Mało Umiarkowanie Mocno

8. W odniesieniu do Twoich oczu i wzroku: Jak bardzo dokucza Ci kurz/pył?

Nie dotyczy

Wcale Mało Umiarkowanie Mocno

9. W odniesieniu do Twoich oczu i wzroku: Jak bardzo przeszkadza Ci zadymione otoczenie?

Nie dotyczy

Wcale Mało Umiarkowanie Mocno

Dziękuję!
